# Cell-Type-Resolved Acetylation Regulator Atlas Defines Immune Endotypes and Druggable Vulnerabilities in Psoriasis

**DOI:** 10.3390/biomedicines14040804

**Published:** 2026-04-01

**Authors:** Mengji Xie, Xiaoxuan Ma, Ying Zhang, Le Kuai, Ying Luo, Jiankun Song, Xiaojie Ding, Yi Ru, Yue Luo, Xiaoya Fei, Seokgyeong Hong, Guoshu Deng, Yonghua Su, Ruiping Wang, Bin Li, Yanwei Xiang, Miao Li, Mi Zhou

**Affiliations:** 1 Department of Dermatology, Yueyang Hospital of Integrated Traditional Chinese and Western Medicine, Shanghai University of Traditional Chinese Medicine, Shanghai 200437, China; m18857542397@163.com (M.X.); max990211@163.com~(X.M.); kuaile@shyueyanghospital.com (L.K.); luoying@shyueyanghospital.com (Y.L.); dingxiaojie1222@163.com (X.D.); hsk2785@hotmail.com (S.H.); dluck0027@163.com (G.D.); suyh2001@shutcm.edu.cn (Y.S.); 2 Institute of Dermatology, Yueyang Hospital of Integrated Traditional Chinese and Western Medicine, Shanghai University of Traditional Chinese Medicine, Shanghai 200437, China; lib@shskin.com; 3 Shanghai Skin Disease Hospital, Institute of Dermatology, School of Medicine, Tongji University, Shanghai 200443, China; lindsey.zhang21@gmail.com (Y.Z.); 18616289432@163.com (J.S.); pansy022@hotmail.com (Y.R.); moon_ms.luo@hotmail.com (Y.L.); xiaoyafei_1994@163.com (X.F.); w19830901@126.com (R.W.); 4 School of Rehabilitation Science, Shanghai University of Traditional Chinese Medicine, Shanghai 201203, China

**Keywords:** psoriasis, lysine acetylation, HDAC, KAT, single-cell RNA-seq, disease endotypes, epigenetic therapy

## Abstract

**Background:** Psoriasis frequently relapses after treatment withdrawal, consistent with persistent epigenetic programs in lesional immune cells. Lysine acetylation is a reversible regulatory layer linking chromatin accessibility, transcription factor activity, and immune-cell effector programs; yet, its cell-type-resolved landscape and clinical stratification value in psoriasis remain incompletely defined. **Methods:** We integrated four bulk transcriptome cohorts of psoriatic and healthy skin (746 psoriasis, 515 controls) with two public skin scRNA-seq datasets. A diagnostic acetylation-regulator signature was derived from 33 curated acetylation regulators, and acetylation endotypes were defined by unsupervised clustering. The cell-type-specific expression was mapped at the single-cell resolution. Key regulators were validated by quantitative real-time polymerase chain reaction (qRT-PCR) in an imiquimod-induced psoriasis-like mouse model, and further verified in an independent dataset (GSE136757). Motif enrichment and drug–target mining were used to prioritize transcriptional regulators and candidate epigenetic therapeutics. **Results:** Sixteen acetylation regulators were differentially expressed in bulk skin, with histone deacetylase (HDAC1) showing the strongest upregulation and lysine acetyltransferase (KAT2A) the strongest downregulation. A 13-gene acetylation signature discriminated psoriasis from controls (area under the curve, AUC 0.886) and separated lesional samples into two acetylation endotypes with divergent pathway states (hypoxia–glycolysis versus oxidative-stress-dominated programs). Single-cell mapping demonstrated immune-restricted acetylation modules, including CREB binding protein (CREBBP)-enriched neutrophils, histone deacetylase 1 (HDAC1)-high cluster of differentiation (CD)8^+^ T cells, and lysine acetyltransferase 6A (KAT6A)/lymphoid enhancer binding factor (LEF1)-enriched CD4^+^ and regulatory T cell (Treg) subsets, coincident with interleukin (IL)-17-related inflammatory programs. In mice, qRT-PCR confirmed the coordinated dysregulation of hub genes and highlighted Hnf1a and Kat6a as reproducible candidates. External validation using the GSE136757 dataset further supports their robust diagnostic performance. Motif analysis nominated interferon regulatory factor (IRF4), YY transcription factor (YY2), and zinc finger protein (ZNF404) as putative transcriptional mediators downstream of acetylation programs, and drug–target mining prioritized epigenetic compounds with subtype-relevant potential, including histone deacetylase (HDAC) inhibitors (e.g., entinostat) and the p300/CREB binding protein (CBP) inhibitor A485. **Conclusions:** This integrative atlas links acetylation regulators to specific immune compartments, defines acetylation endotypes associated with distinct inflammatory programs, and provides a rationale for stratified epigenetic target selection in psoriasis.

## 1. Introduction

Lysine acetylation is a reversible post-translational modification that modulates chromatin accessibility and transcriptional output, thereby shaping immune-cell activation, tolerance, and inflammatory memory [1,2,3]. By balancing the activity of lysine acetyltransferases (KATs), also known as (histone acetyltransferases, HATs) and deacetylases (histone deacetylases, HDACs and sirtuins), acetylation couples environmental and metabolic cues to gene-regulatory programs through both histone and non-histone substrates, including transcription factors and signaling nodes [4]. Because acetylation marks are dynamically written and erased, acetylation regulators represent a druggable layer with the potential to reprogram pathogenic immune states rather than merely suppress downstream cytokines.

Psoriasis is a chronic, immune-mediated inflammatory dermatosis driven by coordinated interactions among keratinocytes, dendritic cells, T cells, and neutrophils, while the interleukin (IL)-23/IL-17 axis is a central pathogenic pathway, and long-lived memory populations, particularly tissue-resident memory T cells (TRM) and other persistent inflammatory cell states, contribute to disease persistence and relapse [5,6]. Although biologics targeting tumor necrosis factor (TNF)-α, IL-17, and IL-23 achieve high clinical response rates, incomplete remission, secondary loss of response, and rapid relapse after treatment withdrawal remains common in real-world practice. These clinical features imply that upstream regulatory layers, including epigenetic programs that stabilize pathogenic recall responses, are incompletely addressed by cytokine blockade alone.

The evidence increasingly supports acetylation as a mechanistic driver of psoriatic inflammation. In T cells, metabolic rewiring can elevate acetyl-CoA availability and promote histone acetylation at the *IL-17*-associated loci, reinforcing Th17 differentiation and amplifying IL-17-mediated pathology [7]. In parallel, inflammatory microenvironments may recruit co-activators such as p300/CREB binding protein (CBP) to acetylate chromatin at hypoxia- or stress-responsive promoters, stabilizing pathogenic effector programs [8]. In the myeloid compartment, previous studies have associated aberrant acetylation regulator activity with chemokine expression and neutrophil recruitment (such as the fact that *KAT8* and *CREBBP* have been shown to increase H4K16ac marks at chemokine *CXCL2* and *CCL3*), consistent with the prominent neutrophilic infiltration and neutrophil extracellular traps (NET)-associated inflammatory amplification in psoriatic plaques [9]. Moreover, multiple studies report the dysregulation of specific HDACs in psoriatic skin, supporting the notion that acetylation regulators may contribute to forming core nodes bridging metabolic adaptation, chromatin remodeling, and effector cytokine production [10]. However, most existing studies focus on individual enzymes in isolated cell types or rely on bulk tissue profiling, which leaves unresolved the cellular compartments in which acetylation programs are most active and clinically relevant.

This limitation is particularly important because psoriatic lesions are highly heterogeneous. Recent single-cell studies have refined the cellular architecture of psoriasis and identified pathogenic dendritic cells (DC) and T-cell states [6,11,12], including inflammatory myeloid subsets that sustain IL-23 production and effector populations such as IL-17-producing CD8^+^ T cells (Tc17) cells [13,14]. Yet, a comprehensive, cell-type-resolved map of acetylation regulators across keratinocytes and immune compartments remains to be systematically characterized [11,12], and it remains unclear whether acetylation profiles can define molecular endotypes with distinct immune–metabolic signaling states. In addition, while acetylation is known to modulate transcription factor activity [15], the downstream acetylation–transcription factor circuitry that rewires psoriatic immunity has not been delineated in a manner that supports rational target prioritization. Finally, although HDAC inhibitors and HAT-directed compounds show preclinical promise [16,17], an acetylation-centered framework that connects cell-type specificity, endotype stratification, and druggability has not yet been developed.

Here, we integrated bulk transcriptomic datasets with single-cell RNA sequencing to construct an acetylation-informed landscape of psoriasis. We derived an interpretable acetylation-regulator signature associated with disease status and mapped acetylation programs to specific immune and stromal compartments at the single-cell resolution, highlighting immune-restricted modules enriched in neutrophils, cluster of differentiation (CD)8^+^ T cells, and CD4^+^ T-cell subsets. We further explored acetylation-linked regulatory wiring using transcription-factor motif enrichment to nominate candidate mediators downstream of acetylation dysregulation, and we applied unsupervised stratification to identify acetylome-driven endotypes with divergent inflammatory and metabolic features. Finally, by integrating protein–protein interactomes with drug–target repositories, we prioritized candidate epigenetic compounds that may be leveraged for subtype-informed therapeutic strategies. Collectively, our study highlights the integration of acetylation-regulator profiling with single-cell mapping, endotype stratification, and drug–target prioritization, providing a framework to support biomarker development and rational epigenetic target selection in psoriasis.

## 2. Materials and Methods

### 2.1. Data Sources

mRNA expression profiles of psoriatic skin lesion samples and healthy skin samples were downloaded from the Gene Expression Omnibus (GEO) database (http://www.ncbi.nlm.nih.gov/geo/, accessed on 1 March 2020; GSE13355, GSE106992, GSE117239, and GSE117468). For genes with multiple probes, median expression values were determined, keeping the probe with the highest mean intensity. Missing values were imputed by the k-nearest neighbors method using the DMwR2 R package (version 0.0.2) [18]. Batch effects were corrected by adjusting the expression values using the ComBat function in the sva R package (version 3.20.0) [19].

### 2.2. Differential Expression of Acetylation Regulators Between Psoriasis and Healthy Samples

The primary source of gene sets used in this study named “Functions and mechanisms of non-histone protein acetylation” (ref. 30467427) was obtained from the National Center for Biotechnology Information (NCBI, https://pubmed.ncbi.nlm.nih.gov/, accessed on 3 March 2020), resulting in the identification of 33 acetylation regulators involved in acetylation modification (Table S1). Additionally, protein–protein interactions (PPIs) were retrieved from the Search Tool for the Retrieval of Interacting Genes/Proteins (STRING) database (https://string-db.org/, accessed on 10 April 2020). The chromosomal locations of the acetylation regulators were mapped using the biomaRt R package (version 2.46.3) and visualized using the circlize package v0.4.10.

The 33 identified acetylation regulators were assessed in the expression matrix of the four merged datasets, and only those represented in the psoriasis datasets were retained for subsequent analyses. To explore the contribution of the acetylation regulators to the pathogenesis of psoriasis, a series of bioinformatics algorithms were used. First, the differentially expressed acetylation regulators between psoriasis and healthy samples were identified using the limma R package (version 3.46.0), based on false discovery rate (FDR) < 0.05. Additionally, pairwise Wilcoxon tests were used to assess differences between psoriasis and healthy controls, adjusting the FDR using the Benjamini–Hochberg procedure.

### 2.3. A Psoriasis Diagnostic Model Based on Acetylation Modification

Univariate logistic regression analysis was performed to identify the potential predictive role of acetylation regulators in psoriasis (*p* < 0.05). Next, using the glmnet R package (version 4.0-2), the acetylation regulators were subjected to feature selection via Least Absolute Shrinkage and Selection Operator (LASSO) regression [20]. Finally, a stepwise multivariate logistic regression analysis was used to determine the acetylation-regulated key genes that can predict psoriasis, i.e., to build a classifier that can effectively distinguish between psoriasis and healthy samples.

A diagnostic model was constructed based on the selected genes, and the risk score for each sample was calculated using the logistic regression coefficients and gene expression values. The accuracy of the diagnostic model was validated by calculating and comparing the risk scores of each individual in the healthy and psoriasis groups. Pairwise correlation analysis was performed among the selected genes, and the variance inflation factors (VIFs) for the final multivariate model were calculated. A VIF value below 5 and a pairwise correlation coefficient |r| < 0.8 [21] indicate a low risk of severe multicollinearity, suggesting that the regression model is stable and reliable. Additionally, a receiver operating characteristics (ROC) analysis was conducted using the pROC R package (version 1.16.2) to further assess the predictive accuracy of the model.

### 2.4. Associations Between Acetylation Regulators and Immune Characteristics in Psoriasis

To analyze the associations of acetylation regulatory factors with human leukocyte antigen (HLA) genes, infiltrating immunocytes, and immune reaction pathways, we first used single-sample gene set enrichment analysis (ssGSEA). The HLA gene set was obtained from previous research [22,23]. The immunocyte infiltration gene sets, involving 28 types of immunocytes, were acquired from previous research [24]; the authors developed gene set variation analysis (GSVA) scores based on the degree of immunocyte infiltration using the GSVA R package (version 1.38.2). The immune reaction pathway gene sets, encompassing 17 immune-related pathways, were downloaded from the Immunology Database and Analysis Portal (ImmPort) database (http://www.immport.org, accessed on 25 April 2026); the researchers computed GSVA scores for the immune-related pathways. The differences in GSVA scores regarding immunocyte infiltration, immune response pathways, and HLA genes between psoriasis and healthy samples were determined using the Wilcoxon test (*p* < 0.05). Additionally, Pearson correlation analysis (*p* < 0.05) was conducted to assess the relationship between acetylation-regulated key genes and HLA genes, immune cells, and immune response pathways.

### 2.5. Quality Control of Single-Cell Datasets

Human psoriasis and healthy skin single-cell RNA sequencing (scRNA-seq) datasets were obtained from GSE220116 and GSE151177. Subsequently, features, barcodes, and matrices were imported into Seurat v.4.5.0 for quality control, dimensionality reduction, cell clustering, and differential analysis of acetylation gene expression. For quality control, the number of gene expressions per cell was maintained between 200 and 2500, while the mitochondrial percentage of each cell was kept below 5%. To eliminate batch effects, the Harmony algorithm was employed for data integration, followed by data normalization and variable feature identification. Over 2000 variable features were identified using the Variance Stabilizing Transformation (VST) method. Principal component analysis (PCA) was utilized for dimensionality reduction, and the ElbowPlot function was employed to visualize the distribution of *p* values, thereby determining the optimal number of principal components. Based on the scree plot analysis, the principal component variance reached a clear plateau around the 40th component. Therefore, the first 40 PCs were retained for downstream clustering and dimensionality-reduction analyses [25,26]. The FindClusters function was executed, and the clustree function was subsequently utilized to determine a resolution of 1.2, which facilitated the separation of cells into unique clusters. Finally, the RunUMAP function was employed for dimensionality reduction, effectively enabling the visualization and exploration of the dataset.

### 2.6. Cell Type Annotation and Acetylation Gene Expression in Single-Cell Clusters

Cell types were annotated based on the expression of canonical marker genes: (1) Keratinocytes: *KRT10*, *KRT2*, *KRT14*, and *KRT5*; (2) Fibroblasts: *DCN* and *COL1A1*; (3) Neutrophils: *S100A8* and *S100A9*; (4) Macrophages: *LYZ* and *CD163*; (5) Natural Killer (NK) cells: *KLRB1* and *GNLY*; (6) Plasma cells: *IGLC2* and *IGKC*; (7) Mature DC: *LAMP3*, *LY75*, *CIITA*, *CD40*, and *HLA-DQA1*; (8) Melanocytes: *DCT*, *TYRP1*, and *MLANA*; and (9) T cells: *CD3D*, *TRAC*, and *TRBC1*. Furthermore, T-cell subtypes were further delineated using specific genes: (1) CD4^+^ T: *CD4*; (2) CD8^+^ T: *CD8A*, and *GZMK*; (3) CD161^+^ T: *KLRB1*; and (4) Regulatory T cells (Treg): *IL2RA*, *FOXP3*, and *CTLA4*. Subsequently, the expression levels of key acetylation genes across various cell types were visualized using dot plots.

### 2.7. Identification of Acetylation Subtypes in Psoriasis

We conducted unsupervised clustering to identify acetylation subtypes among the psoriasis samples based on the expression of the acetylation regulators. The default parameters of the non-negative matrix factorization (NMF) package (version 0.23.0) were applied. The optimal number of clusters was determined based on the number prior to the largest decrease in the cophenetic correlation coefficient. PCA algorithms using acetylation regulator expression data were employed to attempt to distinguish the acetylation subtypes.

### 2.8. Immunological Characteristics of the Two Acetylation Subtypes

In accordance with previous methodologies, the differences in immune characteristics between the two acetylation subtypes were determined using the Wilcoxon test (*p* < 0.05).

### 2.9. The Functions of Psoriasis Acetylation Subtypes

To identify the genes influenced by the acetylation regulators, a differentially expressed genes (DEGs) analysis based on mRNA expression data was carried out between the acetylation subtypes, with FDR < 0.05 and |log_2_(fold change)| > 0.58. The DEGs were then subjected to Gene Ontology (GO) and Kyoto Encyclopedia of Genes and Genomes (KEGG) enrichment analyses using the clusterProfiler R package (version 3.18.1).

The activation status of signaling pathways reflects biological changes. Accordingly, we downloaded the h.all.v7.0.symbols and c2.cp.kegg.v7.0.symbols datasets from the MsigDB database and utilized the GSVA R package (version 1.38.2) to assess the Hallmark and KEGG-related pathways involved in each subtype. Subsequently, we employed the limma R package (version 3.46.0) to identify subtype-specific differences in Hallmark and KEGG-related pathways (FDR < 0.001). Through GSVA analysis, this study unveiled the variability in signaling pathway activation among acetylation subtypes, offering a novel perspective for understanding complex biological processes.

### 2.10. Experimental Validation

#### 2.10.1. Animals and Ethics Approval

The Ethics Committee of Yueyang Hospital affiliated with Shanghai University of Traditional Chinese Medicine approved the animal experiments (No. YYLAC-2020-078-3, Supplementary File S1). All experiments on mice were conducted according to ARRIVE guidelines, and institutional, national, and also European animal regulations. Eight specific-pathogen-free male C57BL/6 mice (6–7 weeks old, weighing 22–25 g) were obtained from Shanghai SLAC Laboratory Animal Co., Ltd. (Shanghai, China). All mice were housed under a 12 h light/12 h dark cycle at a temperature of 21–25 °C, and provided with a standard diet and free access to water.

#### 2.10.2. Psoriasis-like Mouse Model

After shaving the hair of mice in a 2 × 2 cm^2^ square area, the mice were randomly divided into two groups (n = 4): (1) Control group: This group received a topical dose (62.5 mg) of petroleum jelly (Nanchang Baiyun Pharmaceutical Co., Ltd., Nanchang, China; drug approval No. F20050006); and (2) Psoriasis-like mouse model group: This group received a topical dose (62.5 mg) of 5% imiquimod (IMQ) cream (Sichuan Mingxin Pharmaceutical Co., Ltd., Chengdu, China; drug approval No. H20030128). All treatments were applied once daily for a duration of 12 consecutive days [27,28]. The mice were subjected to a 12 h fasting period and provided access to water before sample collection. On day 12, after euthanasia by CO_2_ inhalation, the back tissues of the mice were obtained for further assessment.

#### 2.10.3. qRT-PCR

The skin tissues were homogenized using Bio-Gen PRO200 homogenizer (PRO Scientific Inc., Oxford, CT, USA). TRIzol reagent was used to extract the total RNA (Beyotime, Shanghai China), and an ultraviolet spectrophotometer was used to calculate the concentration and purity. The RNAs were then reverse-transcribed using the PrimeScript™ RT Master Mix (Takara Bio Inc., Kusatsu, Shiga, Japan, Code No. RR036A). quantitative real-time polymerase chain reaction (qRT-PCR) was performed using TB Green Premix Ex Taq™ (Takara, Bio Inc., Kusatsu, Shiga, Japan, Code No. RR420A). The primers utilized for qRT-PCR are listed in Table S2. *p* < 0.05 was deemed significant. The outcome assessor and the data analyst were kept blinded to the treatment groups until the statistical analysis was complete.

### 2.11. External Validation in the GSE136757 Dataset

To evaluate the predictive stability and generalizability of diagnostic model, external validation was performed in the independent dataset GSE136757 [29]. The diagnostic performance of the model was assessed using receiver operating characteristic (ROC) curve analysis, and the area under the curve (AUC) was calculated to quantify predictive accuracy.

### 2.12. Enrichment of Transcription Factors Motifs

Employing R package “RcisTarget” (version 1.10.0), transcription factors were predicted from curated motifs; the normalized enrichment score (NES) of a motif depends on the total number of motifs in the database. Annotations were expanded via smotif similarity and gene sequence. Overexpression of each motif on a gene set was quantified as the AUC for each motif–gene set pair, and NES of each motif was derived from the AUC distribution of all motifs in the gene set.

### 2.13. Identification of Pharmaceutical Targets and Therapeutic Agents for Use in Psoriasis

We derived a human PPI network of psoriasis-related genes from the STRING database, based on a combined score >900. First, using “psoriasis” as the search term, 652 psoriasis-related genes were extracted from the curated set of disease–gene associations in the Phenotype Knowledgebase (phenoPedia) database (https://phgkb.cdc.gov/PHGKB/hNHome.action, accessed on 13 May 2020.), Online Mendelian Inheritance in Man (OMIM) database (http://www.ncbi.nlm.nih.gov/omim, accessed on 14 May 2020), DisGeNET knowledge platform (https://www.disgenet.org/, accessed on 15 May 2020), and Phenolyzer database (https://phenolyzer.wglab.org, accessed on 16 May 2020).

In order to enhance the efficiency of identifying potential therapeutic agents, we queried the pharmGKB platform (www.pharmgkb.org, accessed on 20 May 2020) and DrugBank database (https://www.drugbank.ca, accessed on 20 May 2020). According to the previously described method, the topological proximity between candidate drugs and disease modules was assessed by calculating the shortest path distance between drug targets and psoriasis-associated genes in the PPI network [30]. A shorter network distance indicates closer topological proximity between drug targets and disease-related modules, suggesting greater potential therapeutic relevance [31]. By integrating the density distribution of all drug–disease distances, we adopted distance <5 [30,32] to retain candidate drugs topologically proximate to the psoriasis disease module, with statistical significance further controlled at an FDR < 0.01 [33]. Subsequently, these candidate drugs were mapped to acetylation-related targets based on the acetylation gene set. Removal of entries with proximity = inf and application of the FDR < 0.01 threshold yielded the final set of acetylation-related candidate drugs for subsequent analyses.

## 3. Results

### 3.1. Dysregulated Acetylation Regulators in Psoriasis

**Figure 1:**
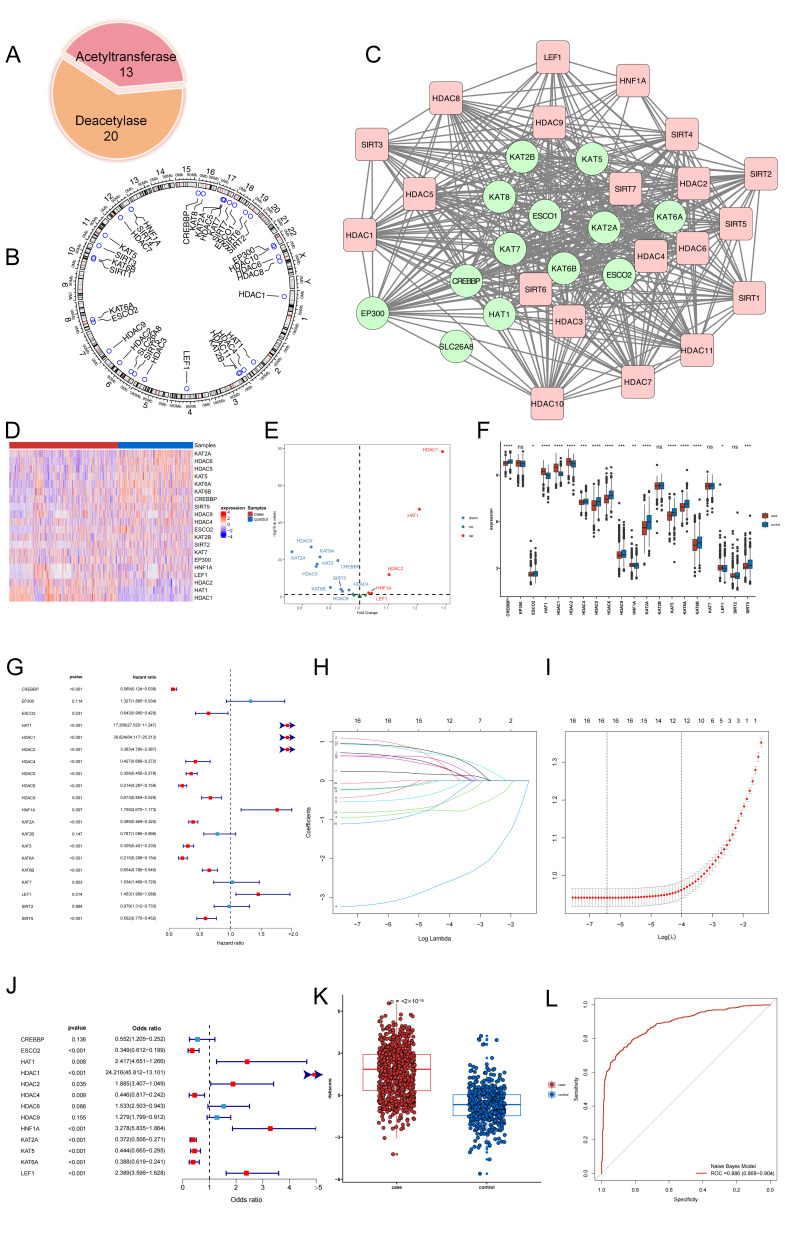
Identify differentially expressed acetylation regulators genes in psoriasis and establish an acetylation diagnostic model that can distinguish between healthy and psoriasis samples. (**A**) The composition summary of acetylation regulators. (**B**) Genomic location and scale bar of 33 acetylation regulators. (**C**) The protein–protein interactions among 33 acetylation regulators. Red blocks indicate the Deacetylase Family, while Green blocks represent the Acetylase Family. (**D**) Heatmap of acetylation mRNA expression. (**E**) The volcano plot shows the summary of expression changing information of acetylation regulators between healthy and psoriasis samples. In red are the upregulated genes, while in blue are the downregulated ones. (**F**) Acetylation expression profile across all psoriasis samples and paired normal tissues. * *p* < 0.05; ** *p* < 0.01; *** *p* < 0.001; **** *p* < 0.0001; ns, not significant difference. (**G**) Univariate logistic regression investigated the relationship between acetylation regulators and psoriasis. Red dots represent *p* < 0.05 while blue points show insignificant differences. (**H**) Least absolute shrinkage and selection operator (LASSO) coefficient profiles. (**I**) 10-fold cross-validation for tuning parameter selection in the LASSO regression. The partial likelihood deviance is plotted against log (λ), where λ is the tuning parameter. Partial likelihood deviance values are shown, with error bars representing SE. The dotted vertical lines are drawn at the optimal values by minimum criteria and 1-SE criteria. (**J**) Distinguishing signature with acetylation RNA regulators was developed by multivariate logistic regression, and the risk scores for psoriasis were calculated. Red dots represent *p* < 0.05, while blue points show insignificant differences. (**K**) The risk distribution between healthy and psoriasis, where psoriasis samples have a much higher risk score than healthy samples. (**L**) The discrimination ability for healthy and psoriasis samples by acetylation regulators was analyzed by receiver operating characteristic (ROC) curve and evaluates by area under the curve (AUC) value.

A total of 33 acetylation regulator genes were retrieved from NCBI (Figure 1A). The positions of these genes were annotated on human chromosomes (Figure 1B). Furthermore, the PPI network revealed that all the acetyltransferases and deacetylases were closely connected (Figure 1C).

After the merging and normalization of the four datasets, the combined dataset comprised 1261 samples, consisting of 746 psoriasis samples and 515 healthy samples. Of the 33 curated acetylation regulators, 20 were retained for downstream analysis after cross-platform probe annotation and the preprocessing of the merged dataset. Among these 20 evaluable regulators, 16 met the differential-expression criterion (FDR < 0.05) between psoriasis and healthy samples (5 upregulated and 11 downregulated) (Figure 1D–F). Among them, the upregulation of the *HDAC1* gene showed the most significant increase, while the downregulation of *KAT2A* was the most pronounced decrease.

### 3.2. The Construction of a Diagnostic Model and Risk-Scoring System

Despite the significant dysregulation of acetylation observed in psoriasis, its potential diagnostic value remains to be explored. Firstly, a univariate logistic regression analysis was conducted to identify acetylation regulators that were associated with psoriasis. The analysis revealed 16 regulators that were statistically significant (*p* < 0.05, Figure 1G). Subsequently, feature selection was performed to select all 16 genes using LASSO regression (Figure 1H,I). In order to further refine the contribution of the aforementioned genes, a multivariate regression-based classifier was developed to distinguish psoriasis samples from healthy samples. This classifier successfully identified 13 genes (*CREBBP*, *ESCO2*, *HAT1*, *HDAC1*, *HDAC2*, *HDAC4*, *HDAC6*, *HDAC9*, *HNF1A*, *KAT2A*, *KAT5*, *KAT6A*, and *LEF1*) through LASSO regression (Figure 1J), indicating their potential roles in the progression of psoriasis. Notably, *HDAC1* emerged as the most probable risk factor. Based on these findings, the risk score is represented as follows: Risk score = −2.141 − 0.594 × (*CREBBP* expression) − 1.052 × (*ESCO2* expression) + 0.883 × (*HAT1* expression) + 3.187 × (*HDAC1* expression) + 0.634 × (*HDAC2* expression) − 0.806 × (*HDAC4* expression) + 0.427 × (*HDAC6* expression) + 0.246 × (*HDAC9* expression) + 1.187 × (*HNF1A* expression) − 0.99 × (*KAT2A* expression) − 0.811 × (*KAT5* expression) − 0.948 × (*KAT6A* expression) + 0.871 × (*LEF1* expression). To assess the impact of multicollinearity on the stability of the final logistic regression model, a pairwise correlation analysis was performed, and all VIF values were below the predefined thresholds (|r| < 0.8, Supplementary Figure S1; VIF < 5, Table S3) [21], indicating a low risk of severe multicollinearity among the predictors. These results support the reliability and stability of the 13-gene classifier.

Thereafter, the psoriasis specimens were found to have a greater risk score in comparison to the healthy specimens (Figure 1K). Moreover, the ROC curve analysis further confirmed the outstanding performance of the risk score in discriminating between psoriasis and healthy samples (area under the ROC curve = 0.886, Figure 1L).

### 3.3. Associations of Acetylation Regulators with Immune Features

**Figure 2:**
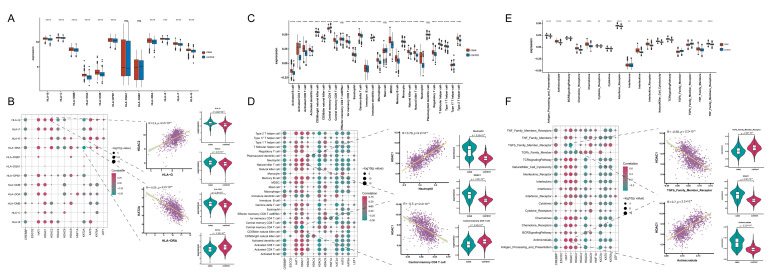
Acetylation regulators and immune characteristics are closely related in psoriasis. (**A**) The expression differences of each HLA gene in acetylation regulators. *** *p* < 0.001; **** *p* < 0.0001; ns, not significant difference. (**B**) The dot-plot demonstrated the correlations between each dysregulated immune HLA cell and each dysregulated acetylation regulator. (**C**) The abundance differences of each immune-microenvironment-infiltrating immunocyte in acetylation regulators. ** *p* < 0.01; **** *p* < 0.0001; ns, not significant difference. (**D**) The dot-plot demonstrated the correlations between each dysregulated immune microenvironment infiltration cell type and each dysregulated acetylation regulator. (**E**) The activity differences of each immune reaction gene-set in acetylation regulators. ** *p* < 0.01; **** *p* < 0.0001. (**F**) The dot-plot demonstrated the correlations between each dysregulated immune pathways and each dysregulated acetylation regulator.

To explore the connections between acetylation regulators and immune characteristics, an analysis was conducted to examine the associations between acetylation regulators and HLA genes, infiltrating immunocytes, and immune response pathways. A comparison of the HLA gene expression in the samples revealed significant differences in the mRNA expression for 10 genes between psoriasis and healthy samples (*p* < 0.05, Figure 2A). Particularly, *HDAC2* was observed a significant positively correlation with *HLA-G* (coefficient = 0.40, *p* < 0.05), while *KAT2A* displayed a significant negative correlation with *HLA-DRA* (coefficient = −0.55, *p* < 0.05) (Figure 2B).

In psoriasis samples, 25/28 immunocytes, including central memory CD4^+^ T cells and neutrophils, displayed differential infiltration patterns compared to healthy samples (*p* < 0.05, Figure 2C). A close relationship between the 13 acetylation regulators and the 28 immunocytes were revealed by correlation analysis. Specifically, *HDAC1* presented the strongest negative correlation with central memory CD4^+^ T cells (coefficient = −0.50, *p* < 0.05), and the strongest positive association with neutrophils (coefficient = 0.79, *p* < 0.05) (Figure 2D).

Lastly, considering the dysregulation of numerous immune reaction pathways in psoriasis, the present study computed GSVA scores for immune-related pathways. Remarkably, significant differences were observed between psoriasis and healthy samples across all 17 immune-related pathways (*p* < 0.05), and correlation analysis revealed that the 13 acetylation regulatory factors are significantly associated with each of these pathways (Figure 2E,F).

### 3.4. Acetylation Regulators Exhibit Distinct Expression Patterns in the Immune Cells of Psoriasis

**Figure 3:**
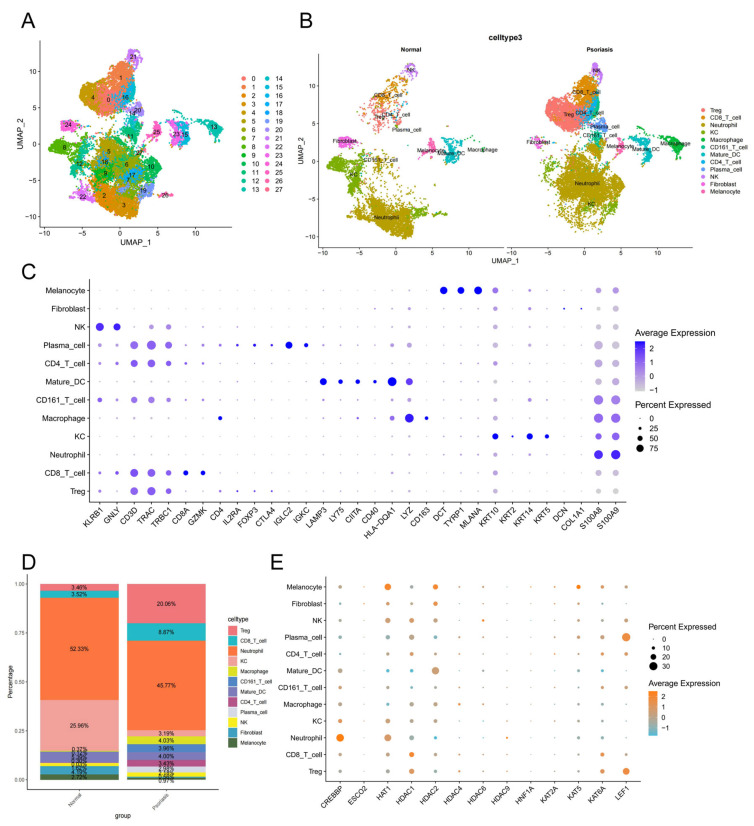
Single-cell sequencing technology unveils distinct expression patterns of acetylation regulators in the immune cells of psoriasis. (**A**) Uniform Manifold Approximation and Projection (UMAP) clustering identified 27 distinct cell clusters. (**B**) Comparison of single-cell clustering between healthy and psoriasis samples. (**C**) Bubble plot visualization of marker gene expression levels in the single-cell dataset. Darker blue indicates higher expression levels, while lighter gray indicates lower expression levels. Larger circles represent a higher proportion of genes expressed within the cell cluster. (**D**) Bar chart showing the proportion of cells in psoriasis and healthy samples. (**E**) Bubble plot visualization of expression levels for 13 key diagnostic genes. Orange indicates higher expression levels, blue indicates lower expression levels, and larger circles represent a higher proportion of genes expressed within the cell cluster.

To elucidate the specific cellular expression differences of acetylation genes in psoriasis, we analyzed scRNA-seq data derived from human psoriatic samples, encompassing skin samples from 24 psoriasis patients and 15 healthy controls. A total of 32,273 cells were retained after quality control. The detailed cell counts for the 15 control samples and 24 psoriasis samples are presented in Supplementary Table S4. Through scRNA sequencing, we successfully identified 12 distinct cell types and their respective marker genes (Figure 3A–C), thereby unraveling the complex heterogeneity of cellular responses in psoriasis.

In psoriasis patients, we observed a significant upregulation in the expression levels of various immune cells (Figure 3D). Through a dot-plot analysis, we found that acetylation genes were expressed across multiple immune cell types (Figure 3E). Notably, *CREBBP* and *HDAC9* were highly expressed in neutrophils; *HDAC1* was highly expressed in CD8^+^ T cells; *HDAC4* exhibited a higher expression in macrophages; *HDAC6* was highly expressed in NK cells; *KAT2A* was highly expressed in CD4^+^ T cells; *KAT6A* was highly expressed in CD8^+^ T cells, Tregs, and CD4^+^ T cells; and *LEF1* was highly expressed in plasma cells and Tregs. These findings indicate that acetylation genes display specific expression patterns in the immune cells of psoriasis, which may be closely related to the onset and progression of the disease.

### 3.5. The Discovery of Acetylation Subtypes in Psoriasis

**Figure 4:**
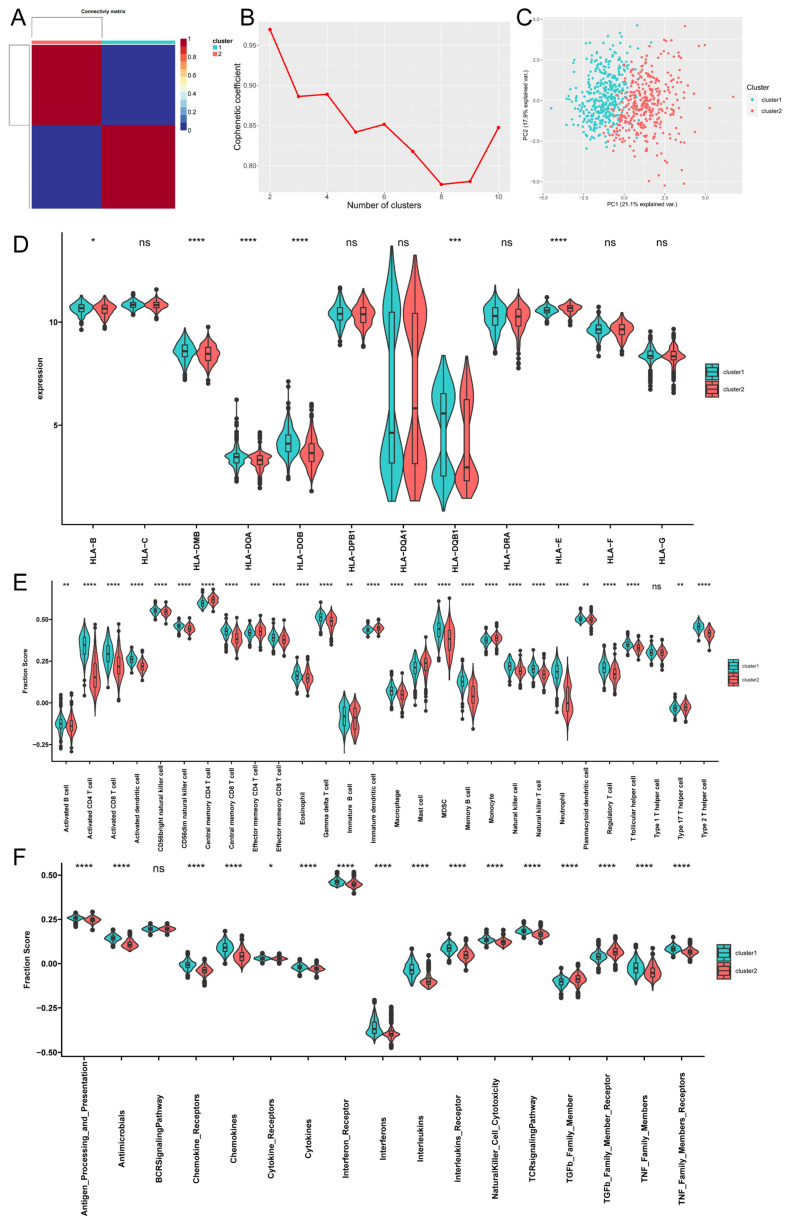
New subtypes of psoriasis discovered based on dysregulated acetylation show expression differences in the immune microenvironment. (**A**) Consensus map of non-negative matrix factorization (NMF) clustering. (**B**) Cophenetic correlation coefficient for clusters k = 2 to k = 10. (**C**) Principal component analysis for the transcriptome profiles of 2 subtypes, showing a remarkable difference on transcriptome between different modification patterns. (**D**) The expression differences of each HLA gene in 2 acetylation modification patterns. (**E**) The abundance differences of each immune-microenvironment-infiltrating immunocyte in 2 acetylation modification patterns. (**F**) The activity differences of each immune reaction gene-set in 2 acetylation modification patterns. * *p* < 0.05; ** *p* < 0.01; *** *p* < 0.001; **** *p* < 0.0001; ns, not significant difference.

Two acetylation subtypes in psoriasis were identified by unsupervised consensus clustering (k = 2) based on the 13 acetylation regulators and 746 psoriasis samples (Figure 4A,B). There were 381 samples in subtype 1 and 365 in subtype 2. The PCA plot indicated a clear differentiation between the subtypes (Figure 4C).

### 3.6. Acetylation Subtypes Exhibit Significantly Distinct Immune Characteristics

In order to elucidate the differences in immune characteristics among various acetylation subtypes, GSVA scores were used to evaluate the expression of HLA genes, the infiltration of immune cells, and the activation of immune response pathways. Notably, six HLA genes showed significant differential expression between the two subtypes (*p* < 0.05, Figure 4D), highlighting a genetic basis for the observed immunological disparities. Further analysis revealed 27 types of immune cells with differential infiltration (*p* < 0.05, Figure 4E) and 16 differential immune-related pathways that were significantly altered (*p* < 0.05, Figure 4F).

Subtype 1 was characterized by a higher degree of immune cell infiltration and a more robust immune response. In contrast, subtype 2 exhibited elevated expression levels in specific immune cell types, including central memory CD4^+^ T cells, effector memory CD4^+^ T cells, mast cells, immature dendritic cells, monocytes, and type 17 T helper cells, compared to subtype 1. Moreover, subtype 2 exhibits higher scores only in TGF-β family members and TGF-β family members’ receptor pathways, suggesting a distinct regulatory mechanism in this subtype. These findings underscore the importance of acetylation modifications in modulating immune characteristics in psoriasis, particularly through the influence on HLA genes, immune cell infiltration, and immune response pathways. The distinct patterns observed in subtype 1 and subtype 2 underscore the heterogeneity in immune responses and the potential for targeted therapeutic interventions based on acetylation subtypes.

### 3.7. Biological Pathways Associated with Acetylation Subtypes

**Figure 5:**
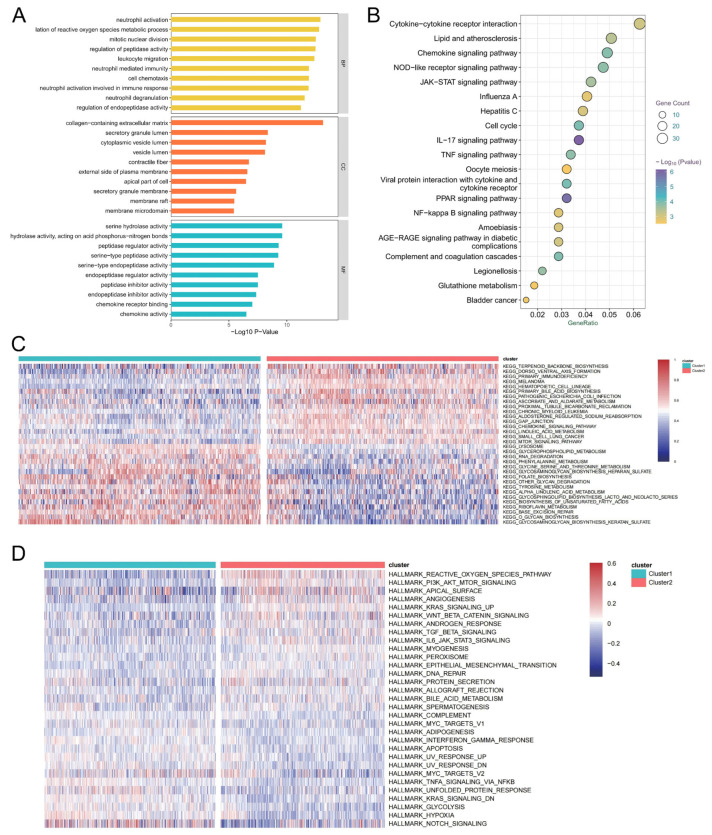
The underlying biological function characteristics diversity among 2 acetylation modification subtypes. The functional annotations and pathway associations of differentially expressed genes between the two subtypes: (**A**) Gene Ontology (GO) enrichment analysis and (**B**) Kyoto Encyclopedia of Genes and Genomes (KEGG) enrichment analysis. The size of the circle represents the number of genes involved, and the abscissa represents the frequency of the genes involved in the term total genes. The differences of KEGG pathway (**C**) and HALLMARKS pathway (**D**) enrichment score between acetylation modification subtype 1 and subtype 2.

In order to explore the molecular mechanisms underlying acetylation-mediated regulation, a comparison of mRNA expression was conducted between the two subtypes, leading to the identification of 1177 DEGs. Subsequently, we analyzed the role of DEGs between the two subtypes in the biologically relevant functions of patients. First, the DEGs were functionally annotated (Figure 5A), the biological processes involved in which mainly include neutrophil activation, leukocyte migration, neutrophil-mediated immunity, neutrophil activation involved in the immune response, and neutrophil degranulation. Second, these DEGs were enriched in the cytokine–cytokine receptor interaction, lipid and atherosclerosis, chemokine signaling pathway, nucleotide-binding oligomerization domain (NOD)-like receptor signaling pathways, and Janus kinase-signal transducer and activator of transcription (JAK-STAT) signaling pathway upon KEGG pathway analysis (Figure 5B).

We further performed GSVA enrichment analyses on the DEGs between the two subtypes, utilizing KEGG and Hallmark pathways. First, a KEGG pathway enrichment analysis identified 126 pathways (FDR < 0.001) (Figure 5C). Subtype 1 exhibited significant differences in various biological processes compared to subtype 2. Notably, it has a high expression of riboflavin metabolism, base excision repair, O glycan biosynthesis, and biosynthesis of unsaturated fatty acids. Conversely, processes such as terpenoid backbone biosynthesis, primary immunodeficiency, and hematopoietic cell lineage were inhibited in subtype 1.

Hallmark pathway enrichment analysis was performed to assess the biological functions of the two subtypes, revealing 31 significantly enriched pathways (FDR < 0.001, Figure 5D). Subtype 1 primarily activates the NOTCH signaling, hypoxia, glycolysis, etc., whereas subtype 2 mainly activates the reactive oxygen species (ROS) pathway, phosphoinositide 3-kinase (PI3K)/protein kinase B (Akt)/mammalian target of rapamycin (mTOR) signaling, angiogenesis, etc. Notably, the Kirsten rat sarcoma viral oncogene homolog (KRAS) signaling pathway exhibited diametrically contrasting alterations between the two subtypes.

### 3.8. Validation of Acetylation Regulator Gene Expression in a Psoriasis-like Mouse Model

**Figure 6:**
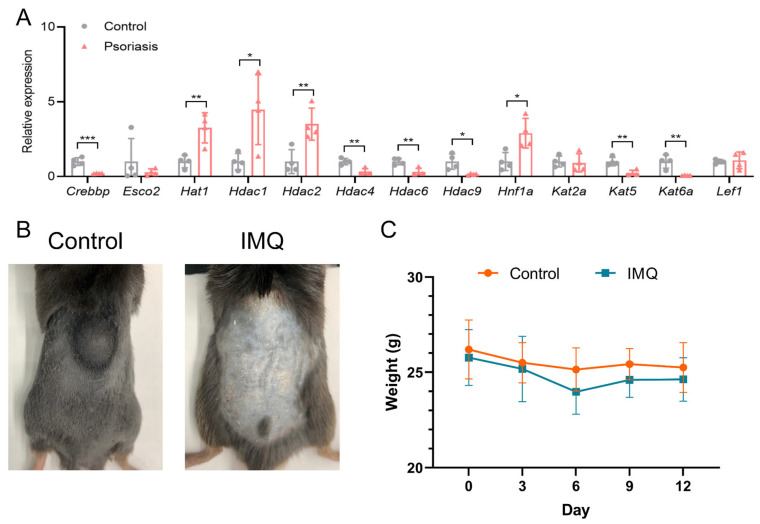
Validation of altered acetylation-regulating gene expression in a mouse model of psoriasis. (**A**) Validation of acetylation-mediated regulators through quantitative real-time polymerase chain reaction (qRT-PCR) experiments in imiquimod (IMQ)-induced psoriasis-like mice compared to the control group (n = 4). * *p* < 0.05; ** *p* < 0.01; *** *p* < 0.001. (**B**) Representative photographs of dorsal skin in the control group and the IMQ-induced model group after 12 days of administration. (**C**) Body weight changes in the control and model groups during the 12-day administration period.

To authenticate the acetylation regulators with a differential expression among psoriasis and healthy samples, qRT-PCR was used to evaluate mRNA expressions in psoriasis-like mouse models and control models. The results indicate that the expression of *Hat1*, *Hdac1*, *Hdac2*, and *Hnf1a* in the psoriasis group was significantly higher compared to the control group, while the expression of *Crebbp*, *Hdac4*, *Hdac6*, *Hdac9*, *Kat5*, and *Kat6a* was significantly decreased in the psoriasis group (Figure 6A). This study innovatively identified *Hnf1a* and *Kat6a* as candidate diagnostic factors, and their differential expression in a psoriasis-like mouse model supported their potential relevance. Additionally, after 12 days of topical administration, mice in the model group exhibited characteristic psoriatic manifestations, including erythema, scaling, and skin thickening on the dorsal skin (Figure 6B), along with a significant decrease in body weight compared with that before administration (Figure 6C). Throughout the experiment, the survival rate of the mice reached 100%.

### 3.9. External Validation of Key Genes

**Figure 7:**
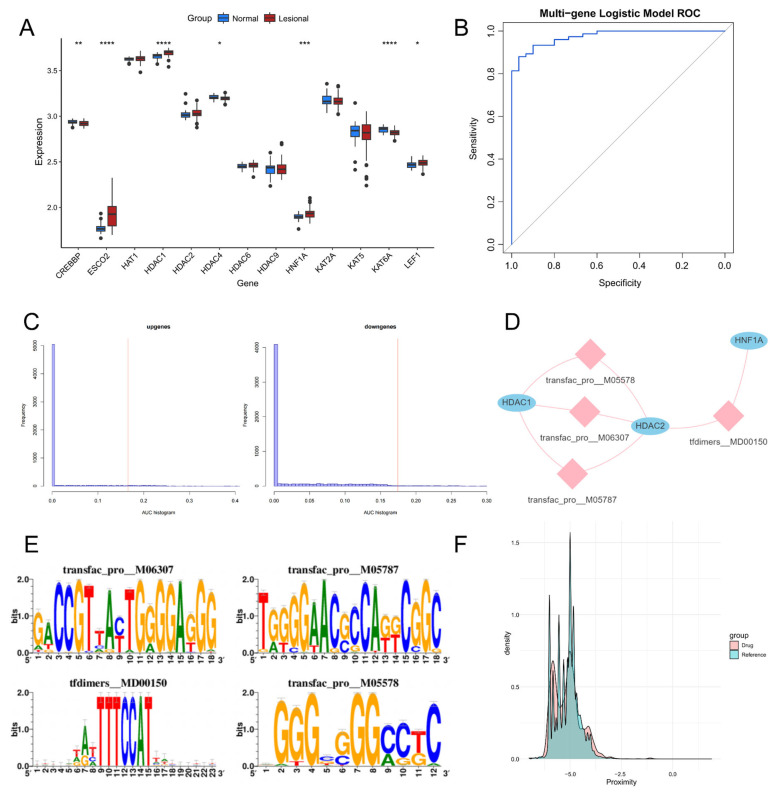
External validation, transcriptional regulatory network, and predicted drug targets of the 13 diagnostic genes. (**A**) Expression patterns of the 13 diagnostic genes in the control group and imiquimod (IMQ)-induced psoriasis model mice. * *p* < 0.05; ** *p* < 0.01; *** *p* < 0.001; **** *p* < 0.0001. (**B**) Overall receiver operating characteristic (ROC) curve analysis of the 13 diagnostic genes (area under the curve (AUC) = 0.974). (**C**) Motif-TF annotation based on normalized enrichment score. The pink vertical lines in the left and right panels represent the AUC values for all upregulated and downregulated genes, respectively. (**D**) Motif-Gene Regulatory Network. The blue nodes represent the key genes, while the pink nodes represent the motifs. (**E**) Motif enrichment and its annotation information. (**F**) Overall distribution of potential and virtual medication.

To further validate the predictive stability of the model, we examined the expression trends of the identified key acetylation genes in an independent external dataset, GSE136757. The key genes exhibited consistent expression trends with those observed in the training set (Figure 7A). ROC curve analysis yielded an AUC of 0.974 for the diagnostic model (Figure 7B), confirming its robust diagnostic performance and stability in predicting psoriasis.

### 3.10. Regulatory Network Analysis of Key Genes

We employed the acetylation genes identified through the aforementioned animal experiments as the gene set for this analysis and found that they are regulated by multiple transcription factors. The results showed that the sequence with the highest normalized enrichment score (NES: 6.73) was annotated as transfac_pro_M06307, and the top-scoring TF motifs included *ZNF404*, *IRF4*, *YY2*, and *ZNF546*. Among all the enriched motifs, the core enriched motifs and their corresponding transcription factors were identified (Figure 7C–E).

### 3.11. Identification of Potential Pharmaceutical Targets and Therapeutic Agents

Psoriasis-related genes were extracted from curated sets of disease–gene associations of various databases, namely, phenoPedia, OMIM, DisGeNET, and Phenolyzer, resulting in the retrieval of 652, 175, 317, and 974 genes, respectively. A total of 974 psoriasis-related genes were yielded for the following analyses by merging the retrieved genes. The PPI network of these psoriasis-related genes, which could represent potential pharmaceutical interventions, consisted of 11,870 nodes and 615,582 edges.

To optimize the efficiency of the discovery of potential therapeutic agents, 1170 agents and 1442 targets were procured from the pharmGKB platform, while 5595 agents and 2747 targets were acquired from the DrugBank database. Subsequently, 6761 agents and 3534 targets were obtained after the amalgamation of the acquired therapeutic agents and pharmaceutical targets (Table S5). Using a previously described method, 679 potential therapeutic agents for treating psoriasis were identified (FDR < 0.01, Figure 7F) based on density curves, with a distance < 5. After hierarchical filtering for acetylation-related targets, 46 candidate drugs were retained. The exclusion of entries with proximity = inf and the application of an FDR < 0.01 significance threshold, 11 acetylation-related candidate drugs were ultimately selected for subsequent functional analyses (Table S6).

## 4. Discussion

Psoriasis is increasingly understood as an epigenetically sustained inflammatory disorder rather than a transient immune activation state. Our integrative analysis suggests that dysregulated lysine acetylation may serve as a central mechanistic axis maintaining pathogenic transcriptional circuits across multiple immune cell lineages. This is consistent with the foundational work establishing acetylation as a major determinant of chromatin accessibility, transcription factor recruitment, and metabolic–epigenetic coupling in immune cells [34]. The coordinated upregulation of deacetylases, particularly *HDAC1*, and concomitant suppression of key acetyltransferases such as *KAT2A*, *KAT5*, *KAT6A*, and *CREBBP* indicate that psoriatic lesions undergo a disease-specific reprogramming of the acetylome. These results align with prior observations that HDACs amplify the inflammatory gene expression in myeloid and lymphoid cells [35,36], whereas aberrant HAT activity reinforces Th17 differentiation and IL-17 transcription [37]. Together, these alterations establish an epigenetic framework that stabilizes pathogenic immune states in psoriasis.

A study found that enhanced keratinocyte glycolysis funnels citrate to acetyl-CoA, escalates H3K9ac and proliferative transcription, and sensitizes mice to IMQ-induced psoriasis; the blockade of this cascade with 2-deoxy-D-glucose (2-DG) or the acetyl-CoA inhibitor 2-hydroxycitrate (2-HC) abolishes epidermal hyperplasia and immune infiltration, establishing the glycolysis–acetylation axis as a primary disease driver [38,39]. *FBP1* loss further amplifies the pathology by leaking mitochondrial ROS, oxidatively inhibiting HDAC2 while augmenting p300 auto-acetylation and global protein acetylation—an “oxidative-stress–acetylation” feed-forward loop quenched in vivo by the ROS scavenger N-acetylcysteine (NAC), which lowers H3K9ac and curtails lesions [40]. Mirroring these findings, our transcriptional stratification segregates psoriasis into two regulatory subtypes: cluster 1 is dominated by the hypoxia–glycolysis module, whereas cluster 2 is governed by oxidative-stress-controlled acetylation, underscoring divergent metabolic–oxidative routes to a shared hyper-acetylated end state.

The single-cell mapping of acetylation regulators provided insight into their cell-type-specific expression patterns, highlighting potential pathogenic roles. Our observation that *CREBBP* is highly expressed in neutrophils complements the emerging evidence that p300/CBP coactivators drive chemokine transcription and NETosis-related inflammatory amplification [41]. Meanwhile, the selective enrichment of *HDAC1* in CD8^+^ T cells is consistent with studies showing that HDAC1 is essential for maintaining effector identity, enforcing transcriptional repression, and controlling cytotoxic T-cell survival [42]. The dysregulated HAT activity within CD4^+^ and CD8^+^ compartments, particularly involving KAT6A, may partly explain the persistent effector cytokine production and metabolic reprogramming, echoing the work demonstrating that the KAT6 family integrates metabolic cues with chromatin remodeling to sustain T-cell activation [43]. Moreover, the enrichment of acetylation-related signatures in CD4^+^ central memory T cells (TCM) supports a growing body of literature indicating that tissue-resident and memory T cells preserve chromatin-encoded inflammatory “memory”, predisposing lesional sites to recurrence [44].

Our transcription factor regulon analysis reveals that IRF4, ZNF404, and YY2 emerged as candidate downstream nodes within the acetylation-driven regulatory network. IRF4, a master regulator of IL-17 production in CD4^+^ T cells and γδT cells, is known to be tightly controlled by acetylation; HDAC11 and HDAC1 directly modulate IRF4 acetylation, affecting its nuclear stability and transcriptional potency [45,46]. These findings provide a potential link between abnormal HDAC activity in psoriatic cells, which may potentiate Th17/Tc17 effector states. Although YY2 remains less characterized, its paralog YY1 has been conclusively shown to recruit both HATs (p300, PCAF) and HDACs to context-dependent loci, with acetylation altering its DNA-binding capacity and transcriptional activity [47]. Our identification of YY2 upstream motifs suggests it may participate in an analogous acetylation-governed regulatory axis contributing to stable inflammatory states.

The translational relevance of our findings is underscored by accumulating evidence supporting acetylation-directed therapeutics. Selective HDAC inhibitors such as entinostat and topical pan-HDAC inhibitors have produced significant reductions in keratinocyte hyperproliferation and IL-17A^+^ γδT-cell infiltration in preclinical psoriasis models [16,17], aligning with our identification of *HDAC1* as a dominant risk gene. Meanwhile, potent p300/CBP inhibitors such as A485 were recently shown to reset pathogenic fibroblast signatures and markedly attenuate imiquimod-induced dermatitis [48], highlighting a feasible route for the epigenetic reprogramming of psoriatic lesions. Beyond symptom reduction, epigenetic therapies offer the unique potential to erase inflammatory memory encoded in keratinocytes and tissue-resident T cells [49], a major contributor to chronicity and relapse. Considering acetylation is reversible and druggable, targeting the acetylome represents a rational strategy to address biologic resistance, disease persistence, and recurrence.

This study also has the following limitations: (1) Our findings are primarily based on mRNA expression levels, and protein-level validation, such as Western blot or immunohistochemistry, is needed to confirm the functional relevance of the identified acetylation regulators. (2) The IMQ-induced mouse model does not fully recapitulate chronic human psoriasis. And in this experiment, the sample size was small (n = 4 per group). (3) During the quality control process for the single-cell RNA sequencing data, while low-quality cells were excluded, we did not perform systematic doublet detection using dedicated algorithms such as DoubletFinder or Scrublet. Future studies incorporating protein-level analyses in larger animal cohorts or human clinical samples, as well as chronic disease models, will be necessary in order to further validate and extend our findings.

## 5. Conclusions

Taken together, our data support acetylation as a potentially important regulatory layer in psoriasis, linking immune activation, transcriptional stability, metabolic reprogramming, and disease heterogeneity in psoriasis. By integrating bulk and single-cell transcriptomics, transcription factor regulomics, and pharmacogenomic screening, we provide a comprehensive framework for understanding acetylation-driven immune dysregulation and nominate actionable epigenetic vulnerabilities. These insights advance the conceptualization of psoriasis as an epigenetically sustained inflammatory disease and lay the foundation for developing acetylation-centered precision therapeutic strategies.

## Data Availability

The mRNA expression data (GSE13355, GSE106992, GSE117239, GSE117468, and GSE136757) supporting this study were sourced from the Gene Expression Omnibus (GEO) database (http://www.ncbi.nlm.nih.gov/geo/, accessed on 1 March 2020). The primary gene set was obtained from the National Center for Biotechnology Information (NCBI, https://pubmed.ncbi.nlm.nih.gov/30467427, accessed on 3 March 2020), and protein–protein interaction data were retrieved from the STRING database (https://string-db.org/, accessed on 10 April 2020). The original data generated from mouse experiments in this study are available upon reasonable request from the corresponding authors, due to the inclusion of preliminary data not yet prepared for public deposition.

## Supplementary Materials

The following supporting information can be downloaded at: https://www.mdpi.com/article/10.3390/biomedicines14040804/s1, File S1: The ARRIVE guidelines 2.0: author checklist; Figure S1: Pairwise correlation analysis of the 13 diagnostic model genes. The absolute values of the correlation coefficients between any two genes were below 0.8, indicating a low risk of severe multicollinearity driven by a single variable; Table S1: List of the 33 acetylation regulators identified based on the gene set from ref. 30467427; Table S2: Primers used for quantitative real-time PCR (qRT-PCR); Table S3: Variance inflation factor (VIF) values for the 13 identified key acetylation-related genes; Table S4: Cell Counts in Each Group After Quality Control; Table S5: Potential drug targets and associated therapeutics; Table S6: The 11 identified acetylation-related candidate drugs.

## Abbreviations and Acronyms

2-DG	deoxy-D-glucose
2-HC	2-hydroxycitrate
AUC	Area Under the recovery Curve
CBP	CREB binding protein
CD	Cluster of Differentiation
DC	Dendritic Cells
DEGs	Differentially Expressed Genes
FDR	False Discovery Rate
GEO	Gene Expression Omnibus
GO	Gene Ontology
GSVA	Gene Set Variation Analysis
HATs	Histone Acetyltransferases
HDACs	Histone Deacetylases
HLA	Human Leukocyte Antigen
IMQ	Imiquimod
JAK-STAT	Janus Kinase-Signal Transducer and Activator of Transcription
KATs	lysine Acetyltransferases
KEGG	Kyoto Encyclopedia of Genes and Genomes
KRAS	Kirsten Rat Sarcoma viral oncogene homolog
LASSO	Least Absolute Shrinkage and Selection Operator
NAC	N-acetylcysteine
NCBI	National Center for Biotechnology Information
NES	Normalized Enrichment Score
NET	Neutrophil Extracellular Traps
NMF	Non-negative Matrix Factorization
NK	Natural Killer
NOD	Nucleotide-binding Oligomerization Domain
PCA	Principal Component Analysis
PI3K/Akt/mTOR	Phosphoinositide 3-kinase/protein kinase B/mammalian target of rapamycin
PPIs	Protein–Protein Interactions
qRT-PCR	quantitative Real-Time Polymerase Chain Reaction
ROC	Receiver Operating Characteristics
ROS	Reactive Oxygen Species
scRNA-seq	single-cell RNA sequencing
ssGSEA	single-sample Gene Set Enrichment Analysis
STRING	Search Tool for the Retrieval of Interacting Genes/Proteins
TCM	Central Memory T Cells
TNF	Tumor Necrosis Factor
Treg	regulatory T cell
TRM	Tissue-resident Memory T Cells
UMAP	Uniform Manifold Approximation and Projection
VIF	Variance Inflation Factors
VST	Variance Stabilizing Transformation
