# Supplementary material for: Cell-Type-Resolved Acetylation Regulator Atlas Defines Immune Endotypes and Druggable Vulnerabilities in Psoriasis
Source: Biomedicines. 2026 Apr 1;14(4):804. doi: 10.3390/biomedicines14040804 (PMC13113943; doi:10.3390/biomedicines14040804)
Supplement: Supplementary file 1 [file biomedicines-14-00804-s001.zip › Supplementary figure.pdf]

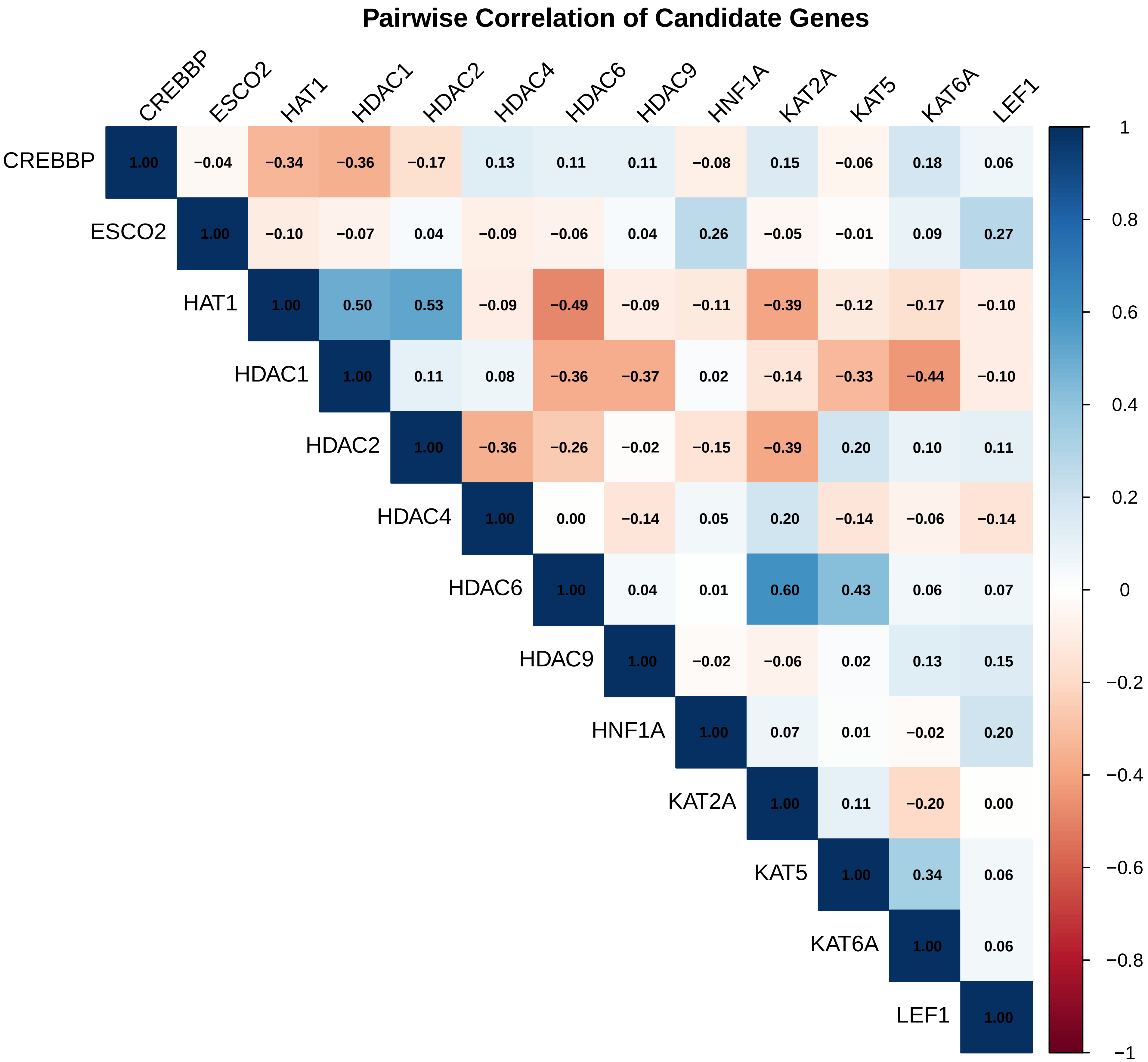

Supplementary Figure S1: Pairwise correlation analysis of the 13 diagnostic model genes. The absolute values of the correlation coefficients between any two genes were below 0.8, indicating a low risk of severe multicollinearity driven by a single variable.
